# Effect of Single and Dual Hydrothermal Treatments on the Resistant Starch Content and Physicochemical Properties of Lotus Rhizome Starches

**DOI:** 10.3390/molecules26144339

**Published:** 2021-07-17

**Authors:** Yun Yeh, Lih-Shiuh Lai

**Affiliations:** Department of Food Science and Biotechnology, National Chung Hsing University, Taichung 40227, Taiwan; lisa840624@gmail.com

**Keywords:** heat-moisture treatment, annealing, resistant starch, thermal properties, pasting properties

## Abstract

Heat-moisture treatment (HMT) changed the morphology and the degree of molecular ordering in lotus rhizome (*Nelumbo nucifera* Gaertn.) starch granules slightly, leading to some detectable cavities or holes near hilum, weaker birefringence and granule agglomeration, accompanied with modified XRD pattern from C- to A-type starch and lower relative crystallinity, particularly for high moisture HMT modification. In contrast, annealing (ANN) showed less impact on granule morphology, XRD pattern and relative crystallinity. All hydrothermal treatment decreased the resistant starch (from about 27.7–35.4% to 2.7–20%), increased the damage starch (from about 0.5–1.6% to 2.4–23.6%) and modified the functional and pasting properties of lotus rhizome starch pronouncedly. An increase in gelatinization temperature but a decrease in transition enthalpy occurred after hydrothermal modification, particularly for hydrothermal modification involved with HMT. HMT-modified starch also showed higher pasting temperature, less pronounced peak viscosity, leading to less significant thixotropic behavior and retrogradation during pasting-gelation process. However, single ANN treatment imparts a higher tendency of retrogradation as compared to native starch. For dual hydrothermally modified samples, the functional properties generally resembled to the behavior of single HMT-modified samples, indicating the pre- or post-ANN modification had less impact on the properties HMT modified lotus rhizome starch.

## 1. Introduction

Starch is a semi-crystalline biopolymer that serves as a storage carbohydrate for energy reserves in many plants, including cereals, roots, tubers, seeds, and fruits. Starch granules in storage tissues can vary in shape, size, amylose/amylopectin ratio and functional properties [1]. Application of native starches in the food industry is kind of limited due to their relatively poor thermal, shear, and acid stability, and also the high rate and extent of retrogradation during storage [2]. However, these disadvantages can be improved by different kinds of modification methods, including physical, chemical and enzymatic methods [3]. Among them, the physical modification of starch by moisture, heat, shear, or radiation has been gaining attention due to the fact that no residues of chemical reagents are present in the modified starch, and can be considered as more natural with higher safety.

**Figure 1:**
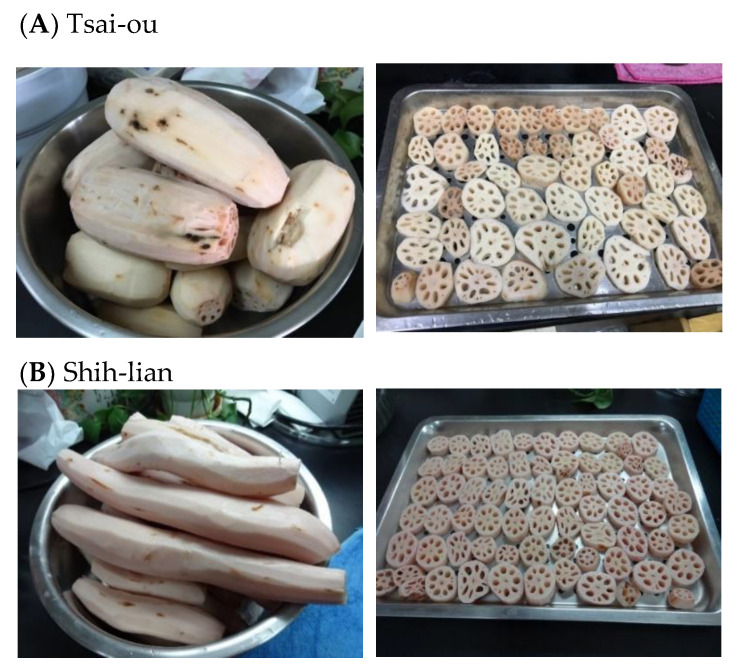
Photos of the two varieties of lotus rhizome (*Nelumbo nucifera* Gaertn.) used in this study from Baihe district, Tainan, Taiwan.

Lotus rhizome (*Nelumbo nucifera* Gaertn.) is an important economic plant widely cultivated in China, India, Japan and Australia [4]. In traditional Asian medicine, the rhizomes and leaves of lotus can be used together with other herbs to treat the fever, sunstroke, diarrhea, dysentery, dizziness, and stomach problems [5]. Lotus rhizome is commercially processed as breakfast, fast food, traditional confectionery and food additives, and is especially suitable for juveniles and seniors [6]. Since the major dry matter of lotus rhizome is starch, it is expected that the eating and nutritional quality of lotus food products will be strongly related to starch properties. Previous studies have reported some of the physicochemical and morphological properties of native lotus rhizome and seed starch [4,6,7,8,9,10]. However, studies on the characteristic changes of lotus rhizome starches by modification are relatively limited, possibly due to the fact that lotus rhizome starch is a relatively exotic source of starch as compared to wheat, corn, tapioca or potato starches [9,10,11]. Hydrothermal treatment is one of the broadly used physical methods to modify the properties of starches [1,2,3]. We assumed that hydrothermal treatments may exert molecular changes of lotus rhizome starch, and modify the rheological properties, including the pasting property and the final viscosity of starch paste during food preparation. Thus, the objective of this study is to investigate the impact of heat-moisture treatment, annealing, and dual hydrothermal modifications on the granule morphology, resistant starch content and functional properties of starches isolated from two common varieties of lotus rhizome harvested in Taiwan, namely Tsai-ou and Shih-lian. As shown in Figure 1, the appearance of Tsai-ou is slightly pinkish, with a larger diameter and shorter length, and is generally consumed by being cooked into dishes. In contrast, Shih-lian appears to be slightly whitish and slender, and is usually processed into lotus rhizome meal, which is basically a flaky powder form of lotus rhizome, and can be consumed by mixing with hot water to make a thickened drink or soup. Understanding the information about property modification by hydrothermal treatment can be useful when applying lotus rhizome starch in various food applications.

## 2. Results and Discussion

### 2.1. Proximate Compositions

Table 1 shows the approximate compositions of lotus rhizome starches isolated from two varieties of lotus rhizome, namely Tsai-ou (abbreviated hereafter as PF due to its pinkish color and fat appearance) and Shih-lian (abbreviated hereafter as WS due to its slightly whitish color and slender appearance). Slight differences in crude lipid, protein and ash contents were observed, possibly due to the variation of plant genetics and cultivation conditions [12]. However, the high N.F.E content in isolated starches from both varieties of lotus rhizome implied high starch purity. 

### 2.2. Granule Morphology

**Figure 2:**
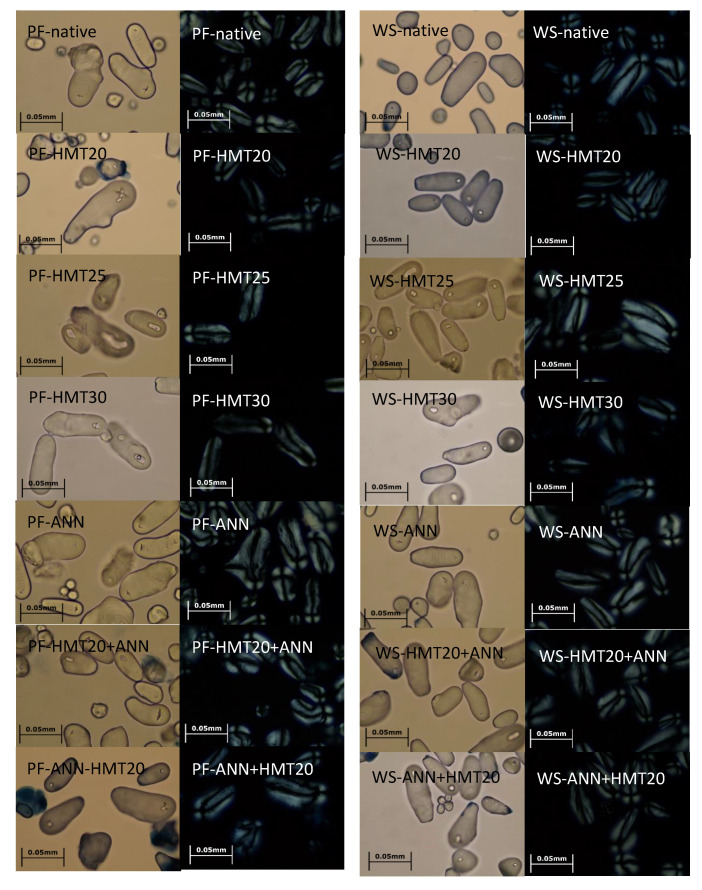
Granule appearance and birefringence photomicrographs of native and modified lotus rhizome starches (400×).

Figure 2 presents the photomicrographs of native and hydrothermally modified lotus rhizome starches from two varieties taken from light and polarized light microscope. It was found that morphology of native PF and WS starches were mostly elongated in shape with relatively larger size, while some granules were spherical in shape with relatively smaller size, which is consistent with the findings from other studies [6,8,13]. Furthermore, the hilum, i.e., the original growing point of lotus rhizome starch, was found to be located at the center (for spherical ones) or near one end of the granules (for elongated ones). By using bacterial α-amylase hydrolysis, Lin et al. [13] also pointed out the hilum of large rhizome starch is asymmetrically located at one end. Under polarized light microscope, all native and hydrothermally modified starch granules retained the characteristic Maltese cross, which reflects the radial arrangement of amylopectin crystallites within the granules, and the hilum was located at the center of the Maltese cross (Figure 2). However, it was also noticed that after HMT, some PF and WS starch granules showed a hole located in the hilum area, and the polarization cross had become somewhat blurry (Figure 1). This is possibly because the high temperature (105 °C) during HMT may increase the mobility of starch chains by thermal energy, resulting in possible rearrangement of the molecular chains at susceptible region (the hilum), and weakened radial orientation of the starch granules [8,14,15]. These changes became more apparent with increasing moisture level during HMT. On the other hand, the morphology of starch granules and its birefringence essentially remained unchanged after ANN, possibly due to the fact that annealing is performed above the glass transition temperature but below the gelatinization temperature of starch in excess water, and the ANN condition applied in this study (50 °C) was quite distant from the onset temperature of starch gelatinization, and the thermal energy of ANN was less than that of HMT. These results are consistent with the findings for annealed wheat, pea, lentil, and navy bean starches [16,17]. For dual hydrothermally modified samples (HMT20 + ANN or ANN + HMT20), the extent of morphological alteration was between that of HMT20 and ANN starches to some extent.

**Figure 3:**
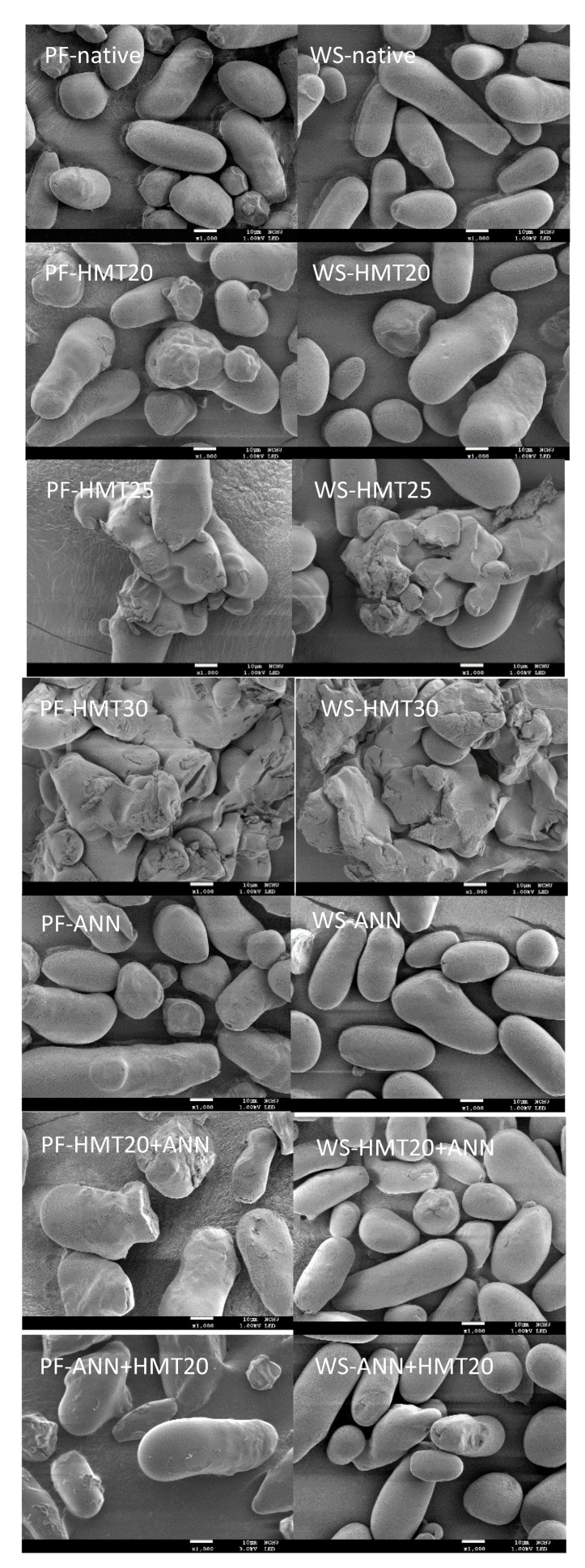
Scanning electron microscopy images of native and modified lotus rhizome starches (1000×).

Figure 3 presented the photomicrographs of native and hydrothermally modified lotus rhizome starches from two varieties taken from scanning electron microscope. Both native lotus rhizome starches showed small rounded and oval-shaped granules with smooth surface, but some dents were observed at one end of the granules, which might be opposite to the hilum location [4]. After HMT and dual hydrothermal modifications, some of the starch granules showed cavities on the surfaces. This might be attributed to the recombination of amylose and amylopectin chains induced by the thermal energy from HMT, making amorphous regions become more compact [18]. In addition, HMT under high moisture level (25 and 30%) resulted in much more granule agglomeration. Granule agglomeration after HMT was also observed in other studies, inferring possibly the occurrence of surface gelatinization of starch granules [19,20,21,22]. On the other hand, ANN had the least impact on the appearance of the granules. Liu et al. [23] also found that HMT-modified buckwheat starch granules exhibited more irregular surfaces and were more aggregated than those of ANN-modified samples.

### 2.3. X-ray Diffraction (XRD) and Relative Crystallinity (RC)

**Figure 4:**
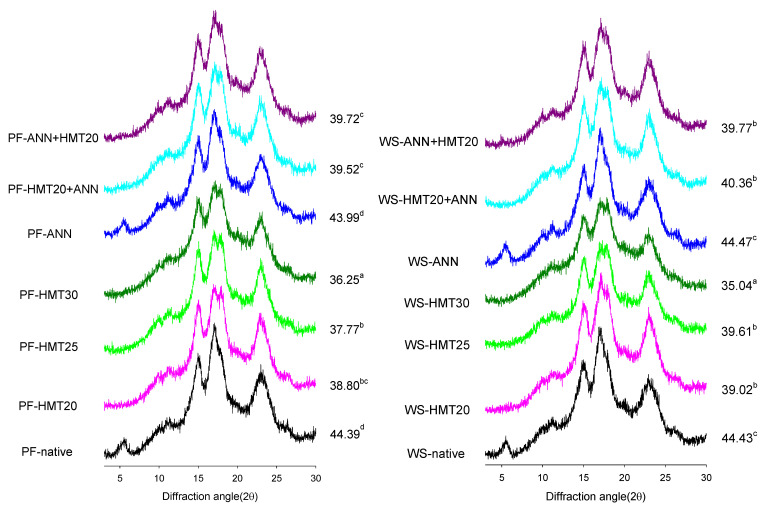
X-ray diffraction patterns and relative crystallinity of native and modified lotus rhizome starches. ^a–d^ Relative crystallinity values with different letter for the same variety are significantly different (*p* < 0.05).

Starch is a semi-crystalline biopolymer, and different crystal phases can lead to different material properties. The crystalline or ordered structures in starch molecules can be investigated generally by X-ray diffraction (XRD) technique in a nondestructive way. Relative crystallinity (RC) can therefore be calculated by the ratio of the integrated diffraction intensity of the crystalline part to that of the amorphous part. X-ray diffraction (XRD) spectra and relative crystallinity (RC) of native/modified lotus rhizome starches were shown in Figure 4. Native lotus rhizome starch exhibited an XRD pattern characterized by the presence of a small peak at 5.6°, strong peaks at 15° and 17°, and a broader peak at 23° 2θ, and can be classified as at C-type allomorph [4,7,8].

After HMT, the peak 2θ at 5.6° (typical for B-type) disappeared and the single peak at 17° split into double peaks at 17° and 18° (typical for A-type), implying the crystalline type of HMT-modified starch transformed from C-type to A-type due to HMT modification. Gunaratne and Hoover [24] suggested that changes in XRD pattern on HMT-modified starches containing B-type unit cells were due to dehydration of the 36 water molecules in the central channel of B-type unit cell and the movement of a pair of double helices into the central channel. Vermeylen, Goderis, and Delcour [25] also suggested that double helical movement during HMT could occur laterally and/or along the vertical axis. Moreover, RC decreased with increasing the moisture content during hydrothermal modification, which is consistent with the findings of other studies on HMT-modified rice and grass pea starches [22,26].

Nevertheless, ANN did not change the XRD pattern and RC of lotus rhizome starches significantly, which is consistent with the findings from other studies on ANN-modified corn and acorn starches [27,28]. Although ANN could increase crystalline perfection, it might not broaden the crystalline region [26]. In addition, thermal energy involved in ANN-modification might be too low to trigger dehydration or movement of the double helices pair [19]. Dual hydrothermal modification also resulted in the transformation of crystalline type from C-type to A-type. However, less impact on RC was shown pre- or post-ANN treatment.

### 2.4. Damage Starch and Amylose Content

Damage starch refers to the small starch fragments or particles broken up from the main starch granules. It may genetically come from starch itself or mechanical disruption during processing such as milling. During dough preparation, appropriate level of damage starch could improve the water absorption and dough mixing properties of flour [29,30]. The amount of damage starch in native lotus rhizome starches was found to be about 1.59% and 0.53% (d.b.), respectively (Table 2). All hydrothermal modification increased the damage starch content of lotus rhizome, but WS starch was less affected as compared to PF starch. SEM results (Figure 2) also revealed that starch granules of WS showed slightly higher surface integrity than PF starch. For HMT-modified samples, the damage starch content generally increased with increasing the moisture content during hydrothermal modification, especially for HMT30 (23.56% and 21.15% for PF and WS, respectively). Similar results have been reported for HMT cassava starch [30]. This is possibly due to the fact that HMT may weaken the radial orientation of starch granules, and the post-modification operations (such as drying and milling) may further increase the level of damage starch [8,14,15,30]. Moreover, although ANN was considered to be a mild hydrothermal modification method, it also increased the level of damage starch significantly. Annealing treatment may facilitate the interaction between amylose–amylose and/or amylose–amylopectin due to the plasticizing effect of water molecules and enhance the crystalline structure perfection; however, it may also create a void or porous structure due to crystalline perfection which allowed more rapid hydrolysis by enzymes [31,32]. Although, under comparable conditions, dual hydrothermally modified samples showed higher damage starch content than single hydrothermally modified ones, the pre-ANN treatment seemed to have some protection for the structural changes by the subsequent HMT20 treatment, as evidenced by a less increase in damage starch content.

The amylose content of native lotus rhizome starches was about 18.38% and 16.43%, respectively (Table 2). The amylose content of lotus rhizome starch increased slightly after HMT and dual hydrothermal modifications, possibly attributed to the cleavage of covalent glycosides linkage by the high thermal energy of HMT, turning the long amylopectin chains into shorter amylopectin chains during HMT process [28,33]. Similarly, WS starch granules were less affected by modifications as compared to PF starch. In contrast, ANN treatment did not significantly affect amylose content. Although the ANN process can rearrange the amylopectin crystalline structure to produce highly ordered regions, it seldom changes the amylose-amylopectin ratio or chain length distribution in most cases [34].

### 2.5. Resistant Starch (RS) Content

Based on the rate of glucose released by amylase and its absorption in the gastrointestinal tract, Englyst, Kingman, and Cummings [35] classified starch into rapidly digestible starch (RDS), slowly digestible starch (SDS), and resistant starch (RS). Among them, RS is defined as “the starch portion that cannot be digested in the small intestine, but is fermented in the large intestine” [36]. The health benefits of RS have been reported as prevention of colonic cancer, hypoglycemic effects, substrate for growth of probiotic microorganism, and inhibition of fat metabolism, etc. [37]. RS content is generally considered to be an index of reduced glycemic response and digestibility. It was found that native WS starch contained significantly higher amount of RS (36.85%) than native PF starch (27.74%) (Table 2). Hydrothermal modification generally significantly decreased the RS, particularly for treatment involved HMT. The influence of ANN on the RS content was less pronounced as compared to HMT. Furthermore, the RS content decreased with increasing moisture content during HMT treatment, and is consistent with the findings of for pea, lentil, navy bean starches and green flour [16,19]. However, the RS content increased after potato, corn, legume, maize and sweet potato starch were modified by HMT or ANN [18,21,36]. This might be due to different starch sources, crystalline degree, amylose content, and amylose/amylopectin interactions.

The amylase digestion rates of starch are related to a barrier that slows down or prevents access or binding of enzyme to starch, and can be linked to the starch structural features that slow down or hamper amylase action as well [38]. Gunaratne et al. [24] thought that crystalline disruption near the granule surface or the number of double helices disrupted in the amorphous regions on HMT could increase the extent of enzyme hydrolysis. Several studies have also shown that A-type crystallites are relatively weaker (due to α(1→6) branch points being present in the crystalline region) than B-type crystallites (due to α(1→6) branch points present solely in the amorphous regions), and hence more susceptible to the attack by amylolytic enzymes [39,40]. These findings are consistent with the XRD results of native (A + B type) and HMT-modified starch (A-type) (Figure 4).

Theoretically, crystalline perfection and amylose-amylose and/or amylose-amylopectin interactions after ANN may increase the RS level, but this was not in agreement with the results in this study. It was suggested that ANN resulted in the slight irreversible swelling of granules, which may account for leaching of some amylose molecules out of the granules, formation of cracks on the granule surface; this change may negate the effect of crystalline perfection and starch chain interactions on enzyme susceptibility [16,19,31]. A similar observation was reported by Chung, Hoover [28] on corn starch. However, because the crystalline type and RC of lotus rhizome starch did not change pronouncedly after ANN, the influence of ANN on the RS content was less pronounced as compared to HMT [19].

### 2.6. Thermal Properties

**Figure 5:**
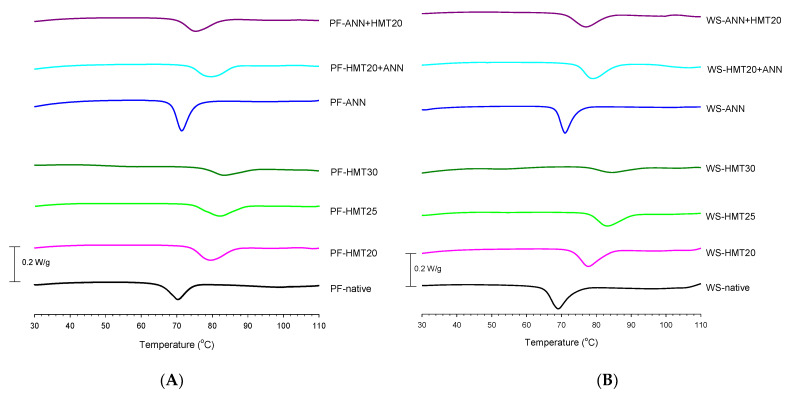
Effect of heat-moisture treatment and annealing on the differential scanning calorimetry of Tsai-ou and Shih-lian starches. (**A**) PF indicates Tsai-ou lotus rhizome starch. (**B**) WS indicates Shih-lian lotus rhizome starch. HMT indicates heat-moisture treatment. The numbers 20, 25 and 30 indicate the moisture levels under HMT. ANN indicates annealing.

The thermal characteristics of starch gelatinization transition were studied by heating them up in the presence of excess water in differential scanning calorimetry (DSC). As shown in Figure 5, the phase-transition-related endothermic changes started to occur at low temperatures, and this process involved a continuous sequence of structural changes, resulting in progressive differences in endothermic patterns from low to high temperatures. It is generally accepted that characteristic temperatures related to gelatinization phenomena (including the onset temperature T_o_, peak temperature T_p_ and conclude temperature T_c_) represent the crystalline stability of starch granules, whereas enthalpy of gelatinization (∆H) reflects the melting of amylopectin crystal fraction with potential contributions from both crystal packing and helix melting enthalpies [41,42]. As shown in Table 3, the gelatinization temperature range of native lotus rhizome starch was 65.05 °C to 74.98 °C, which was within the reported gelatinization range for other lotus starch species (57.35 °C to 82.25 °C) [43]. An increase in T_o_, T_p_, T_c_ and T_c_-T_o_, but a decrease in ∆H, occurred after hydrothermal modification, particularly for hydrothermal modification involved with HMT. The high thermal energy imparted to starch chains by HMT would increase the flexibility of smaller fractions within starch granules, such as the amylose chain fractions and spacers (the segments that link amylopectin double helices to the backbone), and facilitate adjacent double helices interaction via hydrogen bonding. These interactions therefore result in more compact structure and restricted starch chain flexibility in bulk and also in inter-crystalline amorphous regions during granular swelling. Consequently, the enhanced crystalline stability by HMT would require a higher input of thermal energy to incur swelling and the disruption of the crystalline domain during gelatinization (higher T_c_-T_o_) [42]. The higher T_o_ also implies that HMT may partially break the relatively weak crystalline domain of starch granules in advance, thus decreasing the ∆H and RC (as shown in Table 3 and Figure 4) [44]. Furthermore, these changes became more pronounced with increasing the moisture content during HMT.

ANN-modified starches also showed higher T_o_, T_p_ and T_c_, although to a lesser extent than HMT-modification. However, the gelatinization temperature range (T_c_-T_o_) was lower. This is attributed to a possible increase in the amount of ordered double-helices, crystalline perfection, amylose–amylopectin or amylose–amylose interactions, amylose/amylopectin–lipid complex formation, and organized crystalline regions by ANN [18,45]. The reduction in the gelatinization range has also been shown by other research [27,28], indicating that endothermic processes including crystallite melting, swelling, and granules hydration have more homogeneity in ANN-modified starches [46]. Thermal properties of dual-modified starches were similar to those of HMT-modified ones, indicating HMT had a greater effect on sensitive crystallites than did ANN. The gelatinization temperatures of ANN + HMT20 starches were lower than those of HMT20 + ANN starches, but they were still higher than those of ANN starches. Additionally, the gelatinization range and ∆H of dual-modified starches were between those of single-modified ones.

### 2.7. Pasting Properties

Starch applications are ultimately dependent on pasting viscosity. The pasting parameters of native/modified PF and WS starch measured by RVA are presented in Table 4. Compared with native WS starch, native PF starch showed higher peak viscosity and breakdown, which means PF starch had lower stability for heat and shear. After hydrothermal modification, the pasting temperatures of PF and WS starch increased, which was consistent with DSC results (Table 3). Additionally, the peak viscosity, setback and breakdown of HMT-modified PF and WS starch were pronouncedly reduced as compared to those of native starches, implying that HMT modification could enhance the heating, shearing and freezing stability of lotus starches, which would be beneficial for canned, baked and frozen foods [27,28]. Furthermore, the negative breakdown values after HMT also implied the switch from a thixotropic rheological behavior (i.e., a decrease of viscosity under constant shearing) to slightly rheopectic behavior (an increase in viscosity under constant shearing). ANN modification also decreased the peak viscosity and breakdown, but to a lesser extent than HMT. Moreover, ANN also increased the setback slightly. It was assumed that ANN caused the crystalline region to rearrange more perfectly, so the structure of starch molecule could remain stable state after cooling, and showed a greater tendency to retrogradation. However, ANN-modified starch was more resistant to heating and shearing than native starches. For dual hydrothermally modified samples, although there were statistically significant differences between the pasting parameters of HMT20 + ANN and ANN + HMT20, their pasting characteristics resembled the behavior of HMT-modified samples.

## 3. Materials and Methods

### 3.1. Materials

Two common varieties of lotus rhizome harvested in Taiwan, namely Tsai-ou (abbreviated hereafter as PF, due to its pinkish color and fat) and Shih-lian (abbreviated hereafter as WS due to its slightly whitish color and slender appearance), were purchased from a local farmer in Baihe district of Tainan, Taiwan.

### 3.2. Starch Isolation

**Figure 6:**
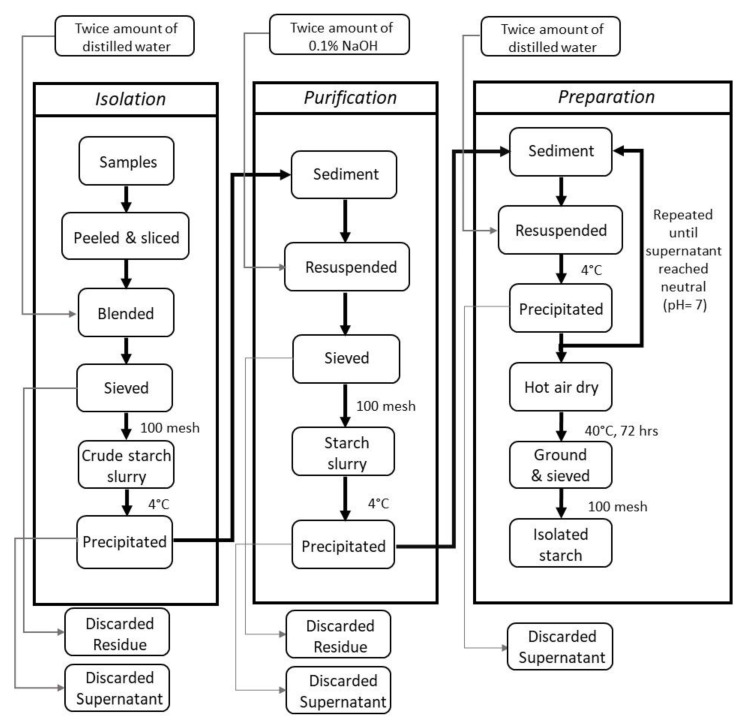
Schematic chart for starch isolation.

The native starch of lotus rhizomes was essentially isolated according to the method described by Rahman, Wheatley, and Rakshit [47]. Figure 6 shows the schematic chart for starch isolation. Briefly, lotus rhizomes were washed, peeled, sliced into small pieces, and homogenized with twice amount of distilled water in a blender (7012S, Waring Commercial Co. LTD., Stamford, CT, USA). The homogenate was filtered with 100-mesh sieves; after that, the filtered starch milk was allowed to sediment overnight (at 4 °C). Subsequently, the supernatant was decanted, and the wet starch was mixed with twice amount of 0.1% NaOH solution. After standing overnight (at 4 °C), the supernatant was decanted again, and the wet starch was mixed with ten times the amount of distilled water by weight. This washing process was repeated several times until the supernatant is neutral (pH = 7). Thereafter, the precipitated starch was dried at 40 °C until the moisture content is less than 10% (d.b.), followed by pulverization and sieving through 100-mesh sieves.

### 3.3. Hydrothermal Treatments

#### 3.3.1. Annealing Treatment (ANN)

Annealing treatment (ANN) was essentially performed based on the method reported by Liu et al. [23]. Starch slurry, obtained by dispersing starch in distilled water (1:4, *w*/*v*), was allowed to equilibrate overnight at 4 °C in sealed containers and incubated at 50 °C for 24 h. After cooling to room temperature, the samples were hot air-dried at 40 °C until the moisture content was less than 10% (d.b.). The annealed starch sample is hereafter referred to as ANN.

#### 3.3.2. Heat-Moisture Treatment (HMT)

Heat-moisture treatment (HMT) was essentially carried out using the method described by Liu et al. [18]. The moisture content of native starch was predetermined, then the moisture levels of starch samples were adjusted to 20, 25, and 30% by adding an appropriate amount of distilled water. Samples were allowed to equilibrate at 4 °C for 24 h in sealed containers, subsequently heated up at 105 °C for 16 h by using a hot-air oven, and then cooled down to room temperature. The HMT samples were then hot air-dried at 40 °C until the moisture content was less than 10% (d.b.). According to the moisture level applied, HMT-modified samples are denoted hereafter as HMT20, HMT25, and HMT30, respectively.

#### 3.3.3. Dual Hydrothermal Modification

Dual hydrothermal modification of lotus rhizome starch was performed by applying HMT20 and ANN treatment in different sequences. For the HMT20 + ANN sample, lotus rhizome starch was subjected to HMT20 treatment (as described in Section 3.3.2), followed by being subjected to ANN treatment (as described in Section 3.3.1). In contrast, for the ANN + HMT20 sample, lotus rhizome starch was subjected to ANN treatment (as described in Section 3.3.1) followed by being subjected to heat-moisture treatment at 20% moisture level (as described in Section 3.3.2).

### 3.4. Proximate Compositions

Standard methods of AOAC [48] were adopted for estimating the moisture (32.1.02), ash (4.1.10), crude lipid (4.5.01), and crude protein content (4.2.03) of lotus rhizome starch. A conversion factor of 6.25 was applied to the total nitrogen content for crude protein estimation. Nitrogen-free extract (N.F.E) content was calculated by the formula of 100% − (%ash + %crude lipid + %crude protein) on a dry basis.

### 3.5. Morphological Observation

#### 3.5.1. Light Micrograph

A drop of iodine solution (0.128 mg/mL I_2_ + 1.28 mg/mL KI) was mixed with an appropriate amount of starch powder on the microscope slide and covered with a coverslip. The morphology and cross birefringence of the starch granules were viewed using an Olympus BX41 light microscope equipped with a simple polarizing attachment (BX-POL, Olympus, Tokyo, Japan) and CCD camera (E330, Olympus, Tokyo, Japan).

#### 3.5.2. Scanning Electron Micrograph

Starch samples were gold-coated using a coater (JEOL-JFC-1600, Auto Fine Coater, Tokyo, Japan) then examined using a scanning electron microscope system (JEOL-JSM6700F, Tokyo, Japan) under 3 kV.

### 3.6. Crystallinity by X-ray Diffraction (XRD)

Crystallinity of native and hydrothermally modified starch samples was analyzed using a Powder X-ray diffractometer (X’Pert Pro MRD, PAnalytical, Netherlands, Holland) operating at 45 kV and 40 mA. Starch sample was equilibrated over a saturated NaCl solution at room temperature (RH = 75%) for one week before analysis [49]. The XRD pattern was obtained from 3° to 50° (2θ) at a scanning speed of 2θ min^−1^ and a step size of 0.02°. The relative crystallinity was quantified as the ratio of the crystalline area to the total area between 3° and 50° (2θ) using the PeakFit software (v4.00, 1995, Jandel Scientific Software, AISN Software Inc, Erkrath, Germany).

### 3.7. Damage Starch and Amylose Content

Damage starch and amylose content were determined according to the AACC method 76-31.01 [50] using a starch damage assay kit and an amylose reagent kit (K-AMYL 12/16) (Megazyme International Ireland Ltd. Co., Wicklow, Ireland), respectively.

### 3.8. Resistant Starch (RS) Content

The content of resistant starch (RS) of native and hydrothermally modified starch samples were determined according to the analysis procedure provided by the Resistant Starch Assay Kit (Megazyme International Ireland Ltd. Co., Wicklow, Ireland).

### 3.9. Thermal Properties by Differential Scanning Calorimetry (DSC)

The thermal properties of native and hydrothermally modified starches were measured using differential scanning calorimetry (DSC822, Mettler Toledo, Switzerland) according to the method of Man et al. [8]. Starch sample was precisely weighed (2.5 mg, d.b.) and mixed with 3 times (by weight) deionized-distilled water (total weight = 10 mg). The mixture was hermetically sealed in an aluminum pan and kept overnight at 4 °C for moisture equilibration, then equilibrating for 1 h at room temperature prior to thermal analysis. Thermal properties of starch sample were measured using a differential scanning calorimetry by heating from 25 to 110 °C at a rate of 10 °C/min. The onset, peak and concluding temperature, as well as enthalpy during phase transition were then determined.

### 3.10. Pasting Properties by Rapid Visco Analysis (RVA)

A Rapid Visco Analyzer RVA-Ezi (Newport Scientific Pty. Ltd., Warriewood, NSW, Australia) was used to determine the pasting properties of native and hydrothermally modified starches. The starch sample (1.68 g, d.b.) was weighed into the RVA canister and water was added to make a total sample weight of 28 g. The following heating/cooling profiles were used: holding at 50 °C for 1 min, heating to 95 °C in 3.5 min, holding at 95 °C for 3 min, subsequently cooling to 50 °C in 3.5 min, and holding at 50 °C for 3 min. A constant paddle rotating speed (160 rpm) was used throughout the whole experiment except for a speed of 960 rpm for the first 10 s to disperse the sample. Viscosity profiles of lotus rhizome starches during the heating/cooling procedure were recorded.

### 3.11. Statistical Analysis

All experiments were conducted in triplicate, and data are expressed as the means ± standard deviation. SPSS software (Version 19.0, IBM Corp., Armonk, NY, USA, 2010) was used to carry out the one-way analysis of variance (ANOVA), and the post hoc analysis applied was Duncan’s test. Significant differences between means were determined at a confidence interval of 95% (*p* < 0.05).

## 4. Conclusions

Among the two lotus rhizome varieties studied, the functional properties of WS lotus rhizome starches were less affected by modifications than the PF lotus rhizome starch. As compared to ANN, HMT and dual hydrothermal modifications changed the starch properties to a greater degree. This suggests that crystallite disruption in HMT starch has a greater impact on starch properties than crystalline perfection in ANN starch. The results showed that HMT and dual modification were more effective in increasing thermal/shearing stability and decreasing the extent of setback, implying suitability for canned, baked and frozen foods. Although the ANN-modified sample did not show pronounced improvement in thermal/shearing stability, it had an adequate damage starch content, possessing the potential to be a flour treatment agent for the purpose of adjusting dough mixing properties. Moreover, hydrothermal modification techniques decrease the resistant starch content of lotus rhizome starch to varying degrees, and could be considered in order to adjust the digestibility of starch-based foods. This information could be useful for applications of lotus rhizome starch in various food preparations.

## Figures and Tables

**Table 1:** Proximate compositions of lotus rhizome starches on dry basis ^2^.

Sample	Crude Lipid	Crude Protein	Ash	N.F.E ^3^
	(%, d.b.)			
PF starch ^1^	0.03 ± 0.00	0.91 ± 0.13	0.08 ± 0.01	98.99 ± 0.13
WS starch	0.11 ± 0.02	0.21 ± 0.03	0.04 ± 0.01	99.65 ± 0.01

^1^ PF indicates Tsai-ou lotus rhizome. WS indicates Shih-lian lotus rhizome. ^2^ Each data was expressed as the mean of three replications ± standard deviation. ^3^ Nitrogen-free extract (N.F.E) = 100 − (crude lipid content + crude protein content + ash content).

**Table 2:** Starch compositions of native/modified lotus rhizome starches on dry basis ^2^.

Sample Code ^1^	Damage Starch	Amylose	Resistant Starch
	(%, d.b.)		
PF-native	1.59 ± 0.02 ^a^	18.38 ± 0.03 ^ab^	27.74 ±0.15 ^f^
PF-HMT20	2.53 ± 0.15 ^b^	18.79 ± 0.58 ^abc^	8.29 ± 0.14 ^d^
PF-HMT25	3.40 ± 0.04 ^c^	19.25 ± 0.87 ^bcd^	5.40 ± 0.11 ^b^
PF-HMT30	23.56 ± 0.61 ^g^	19.52 ± 0.28 ^cd^	2.69 ± 0.04 ^a^
PF-ANN	6.19 ± 0.00 ^d^	18.03 ± 1.01 ^a^	20.02 ± 0.86 ^e^
PF-HMT20 + ANN	8.65 ± 0.12 ^f^	20.02 ± 0.27 ^d^	7.11 ± 0.22 ^c^
PF-ANN + HMT20	7.11 ± 0.21 ^e^	19.29 ± 0.31 ^bcd^	3.35 ± 0.09 ^a^
WS-native	0.53 ± 0.01 ^a^	16.43 ± 0.45 ^a^	35.39 ± 0.56 ^d^
WS-HMT20	2.39 ± 0.06 ^b^	17.50 ± 0.85 ^ab^	5.57 ± 0.06 ^b^
WS-HMT25	3.23 ± 0.05 ^c^	20.90 ± 0.27 ^d^	3.35 ± 0.05 ^a^
WS-HMT30	21.15 ± 0.35 ^f^	19.97 ± 0.87 ^d^	2.68 ± 0.10 ^a^
WS-ANN	3.07 ± 0.11 ^c^	17.46 ± 0.81 ^ab^	16.52 ± 1.43 ^c^
WS-HMT20 + ANN	6.29 ± 0.03 ^e^	17.74 ± 0.52 ^bc^	5.73 ± 0.05 ^b^
WS-ANN + HMT20	3.80 ± 0.11 ^d^	18.59 ± 0.27 ^c^	5.17 ± 0.07 ^b^

^1^ PF indicates Tsai-ou lotus rhizome starch. WS indicates Shih-lian lotus rhizome starch. HMT indicates heat-moisture treatment. 20, 25 and 30 indicate the moisture level under HMT. ANN indicates annealing. ^2^ All data are expressed as the mean of three replications ± standard deviation. ^a–g^ Means with different letter within the same column for the same variety are significantly different (*p* < 0.05).

**Table 3:** Differential scanning calorimetry parameters of native/modified lotus rhizome starches ^3^.

Sample Code ^1^	T_o_ (°C) ^2^	T_p_ (°C)	T_c_ (°C)	T_c_-T_o_ (°C)	∆H (J/g)
PF-native	65.84 ± 0.19 ^a^	70.04 ± 0.15 ^a^	74.98 ± 0.27 ^a^	9.15 ± 0.20 ^b^	3.96 ± 0.09 ^d^
PF-HMT20	73.06 ± 0.59 ^d^	79.62 ± 0.37 ^d^	86.12 ± 0.15 ^c^	13.07 ± 0.58 ^c^	3.30 ± 0.16 ^c^
PF-HMT25	74.74 ± 0.05 ^f^	81.49 ± 0.26 ^e^	88.24 ± 0.29 ^e^	13.50 ± 0.25 ^d^	2.91 ± 0.08 ^b^
PF-HMT30	77.72 ± 0.79 ^g^	82.67 ± 0.50 ^f^	90.72 ± 0.75 ^f^	13.00 ± 0.04 ^cd^	1.44 ± 0.02 ^a^
PF-ANN	68.47 ± 0.16 ^b^	71.38 ± 0.26 ^b^	75.08 ± 0.46 ^a^	6.60 ± 0.30 ^a^	3.83 ± 0.25 ^d^
PF-HMT20 + ANN	73.86 ± 0.21 ^e^	79.35 ± 0.21 ^d^	86.77 ± 0.64 ^d^	12.91 ± 0.56 ^cd^	3.36 ± 0.38 ^c^
PF-ANN + HMT20	70.23 ± 0.14 ^c^	75.11 ± 0.02 ^c^	82.98 ± 0.45 ^b^	12.75 ± 0.33 ^c^	3.35 ± 0.21 ^c^
WS-native	65.05 ± 0.18 ^a^	68.82 ± 0.21 ^a^	73.80 ± 0.29 ^a^	8.75 ± 0.13 ^b^	4.09 ± 0.20 ^d^
WS-HMT20	73.32 ± 0.18 ^d^	77.69 ± 0.21 ^d^	83.90 ± 0.16 ^c^	10.58 ± 0.09 ^c^	3.34 ± 0.30 ^c^
WS-HMT25	78.44 ± 0.27 ^f^	83.21 ± 0.21 ^f^	90.10 ± 0.53 ^e^	11.66 ± 0.64 ^d^	2.79 ± 0.26 ^b^
WS-HMT30	78.41 ± 1.00 ^f^	84.81 ± 0.98 ^g^	93.78 ± 1.46 ^f^	15.37 ± 0.47 ^e^	1.60 ± 0.06 ^a^
WS-ANN	68.18 ± 0.01 ^b^	70.76 ± 0.01 ^b^	74.42 ± 0.10 ^a^	6.24 ± 0.10 ^a^	4.06 ± 0.13 ^d^
WS-HMT20 + ANN	74.39 ± 0.06 ^e^	78.80 ± 0.10 ^e^	85.18 ± 0.29 ^d^	10.92 ± 0.18 ^c^	3.48 ± 0.25 ^c^
WS-ANN + HMT20	71.13 ± 0.33 ^c^	76.76 ± 0.04 ^c^	83.16 ± 0.02 ^b^	12.03 ± 0.35 ^d^	3.25 ± 0.17 ^c^

^1^ PF indicates Tsai-ou lotus rhizome starch. WS indicates Shih-lian lotus rhizome starch. HMT indicates heat-moisture treatment. 20, 25 and 30 indicate the moisture level under HMT. ANN indicates annealing. ^2^ To indicates onset temperature. T_p_ indicates peak temperature. T_c_ indicates conclusion temperature. T_c_-T_o_ indicates gelatinization temperature range. ∆H indicates gelatinization enthalpy. ^3^ Each data was expressed as the mean of three replications ± standard deviation. ^a–g^ Means with different letter within the same column for the same variety are significantly different (*p* < 0.05).

**Table 4:** Rapid-visco parameters of native/modified lotus rhizome starches ^2^.

Sample Code ^1^	Peak Time (min)	Pasting Temperature (°C)	Peak Viscosity	Breakdown	Holding Strength	Setback	Final Viscosity
			(cP)				
PF-native	4.10 ± 0.10 ^a^	75.53 ± 0.04 ^a^	2844.54 ± 23.34 ^f^	1025.46 ± 2.12 ^e^	1818.96 ± 25.46 ^f^	663.00 ± 16.97 ^e^	2481.96 ± 8.49 ^e^
PF-HMT20	5.87 ± 0.19 ^bc^	82.33 ± 0.04 ^c^	259.00 ± 5.66 ^b^	−5.00 ± 4.24 ^c^	264.00 ± 1.41 ^b^	167.00 ± 5.66 ^a^	431.00 ± 7.07 ^a^
PF-HMT25	6.00 ± 0.00 ^c^	85.73 ± 0.53 ^d^	407.33 ± 2.08 ^c^	−49.33 ± 1.53 ^a^	456.67 ± 2.52 ^d^	212.00 ± 5.00 ^b^	668.67 ± 7.51 ^c^
PF-HMT30	6.00 ± 0.00 ^c^	86.58 ± 0.04 ^e^	710.00 ± 7.07 ^d^	−24.5 ± 0.71 ^b^	734.50 ± 6.36 ^e^	302.00 ± 5.66 ^d^	1036.50 ± 12.02 ^d^
PF-ANN	5.70 ± 0.10 ^b^	75.80 ± 0.07 ^a^	2121.00 ± 29.70 ^e^	149.50 ± 12.02 ^d^	1971.50 ± 17.68 ^g^	771.50 ± 30.41 ^f^	2743.00 ± 48.08 ^f^
PF-HMT20 + ANN	5.97 ± 0.00 ^c^	81.85 ± 0.64 ^c^	184.00 ± 2.83 ^a^	−42.50 ± 0.71 ^a^	226.50 ± 3.54 ^a^	169.00 ± 4.24 ^a^	395.50 ± 0.71 ^a^
PF-ANN + HMT20	5.97 ± 0.00 ^c^	80.60 ± 0.00 ^b^	270.50 ± 7.78 ^b^	−44.00 ± 9.90 ^a^	314.50 ± 2.12 ^c^	259.50 ± 3.54 ^c^	574.00 ± 1.41 ^b^
WS-native	4.37 ± 0.05 ^a^	74.35 ± 0.50 ^a^	2614.98 ± 29.36 ^e^	745.00 ± 19.11 ^e^	1869.98 ± 25.62 ^d^	698.99 ± 28.08 ^c^	2569.03 ± 16.06 ^e^
WS-HMT20	6.00 ± 0.00 ^d^	83.50 ± 0.00 ^d^	147.00 ± 1.41 ^a^	−25.50 ± 0.71 ^ab^	172.50 ± 2.12 ^ab^	90.50 ± 3.54 ^ab^	263.00 ± 1.41 ^ab^
WS-HMT25	5.97 ± 0.00 ^cd^	87.03 ± 0.11 ^e^	200.50 ± 7.78 ^b^	−2.50 ± 3.54 ^bc^	203.00 ± 4.24 ^b^	74.50 ± 3.54 ^a^	277.50 ± 7.78 ^b^
WS-HMT30	5.92 ± 0.02 ^bc^	88.33 ± 0.04 ^f^	376.50 ± 0.71 ^c^	2.00 ± 2.00 ^c^	374.50 ± 3.54 ^c^	129.50 ± 13.44 ^b^	504.00 ± 16.97 ^d^
WS-ANN	5.85 ± 0.03 ^b^	75.43 ± 0.11 ^b^	2093.00 ± 38.18 ^d^	57.50 ± 7.78 ^d^	2035.50 ± 30.41 ^e^	861.00 ± 39.60 ^d^	2896.50 ± 9.19 ^f^
WS-HMT20 + ANN	5.97 ± 0.00 ^cd^	83.48 ± 0.11 ^d^	116.50 ± 3.54 ^a^	−21.00 ± 1.41 ^abc^	137.50 ± 4.95 ^a^	101.50 ± 10.61 ^ab^	239.00 ± 15.56 ^a^
WS-ANN + HMT20	5.99 ± 0.02 ^cd^	81.88 ± 0.04 ^c^	146.50 ± 10.61 ^a^	−41.50 ± 0.71 ^a^	188.00 ± 11.31 ^b^	121.00 ± 2.83 ^ab^	309.00 ± 14.14 ^c^

^1^ PF indicates Tsai-ou lotus rhizome starch. WS indicates Shih-lian lotus rhizome starch. HMT indicates heat-moisture treatment. 20, 25 and 30 indicate the moisture level under HMT. ANN indicates annealing. ^2^ All data are expressed as the mean of three replications ± standard deviation. ^a–g^ Means with different letter within the same column for the same variety are significantly different (*p* < 0.05).

## Data Availability

The data presented in this study are available on request from the corresponding author. The data are not publicly available due to ethical restriction and the intellectual property issue.
